# Production of a recombinant vaccine candidate against *Burkholderia pseudomallei* exploiting the bacterial *N*-glycosylation machinery

**DOI:** 10.3389/fmicb.2014.00381

**Published:** 2014-07-29

**Authors:** Fatima Garcia-Quintanilla, Jeremy A. Iwashkiw, Nancy L. Price, Chad Stratilo, Mario F. Feldman

**Affiliations:** ^1^Department of Biological Sciences, University of AlbertaEdmonton, AB, Canada; ^2^Defence Research and Development Canada – Suffield Research CentreMedicine Hat, AB, Canada

**Keywords:** glycobiology, vaccines, protein glycosylation, microbiology and biotechnology, molecular biology, mass spectrometry

## Abstract

Vaccines developing immune responses toward surface carbohydrates conjugated to proteins are effective in preventing infection and death by bacterial pathogens. Traditional production of these vaccines utilizes complex synthetic chemistry to acquire and conjugate the glycan to a protein. However, glycoproteins produced by bacterial protein glycosylation systems are significantly easier to produce, and could possible be used as vaccine candidates. In this work, we functionally expressed the * Burkholderia pseudomallei* O polysaccharide (OPS II), the * Campylobacter jejuni* oligosaccharyltransferase (OTase), and a suitable glycoprotein (AcrA) in a designer *E. coli* strain with a higher efficiency for production of glycoconjugates. We were able to produce and purify the OPS II-AcrA glycoconjugate, and MS analysis confirmed correct glycan was produced and attached. We observed the attachment of the *O*-acetylated deoxyhexose directly to the acceptor protein, which expands the range of substrates utilized by the OTase PglB. Injection of the glycoprotein into mice generated an IgG immune response against *B. pseudomallei*, and this response was partially protective against an intranasal challenge. Our experiments show that bacterial engineered glycoconjugates can be utilized as vaccine candidates against *B. pseudomallei*. Additionally, our new *E. coli* strain SDB1 is more efficient in glycoprotein production, and could have additional applications in the future.

## INTRODUCTION

*Burkholderia pseudomallei*, a Gram-negative saprophyte, is the causative agent for melioidosis and is endemic in Southeast Asia and Northern Australia (Cheng and Currie, 2005). It is highly resistant to harsh environmental pressures, and it is classified as a potential class B bioterrorism weapon due to its high infectivity when aerosolized (Silva and Dow, 2013). Several virulence factors have been identified, including multiple Type III and VI secretion systems, toxins, capsular polysaccharide, and lipopolysaccharide (LPS; Nandi and Tan, 2013). Two different LPS structures named O-polysaccharide (OPS) I and II are present in *B. pseudomallei*, and OPS II was shown to be required for serum resistance and virulence (Knirel et al., 1992; Perry et al., 1995; DeShazer et al., 1998).* B. pseudomallei* has an intrinsically high resistance to several different classes of antibiotics, which increases the potential danger of this organism. Due to the increasing prevalence of new resistance genes, and the increasing number of cases, new alternatives to treat and prevent melioidosis are required.

Immunization is one of the best available tools against infection, and it is significantly more cost effective than treatment after disease has occurred. Three main classes of vaccines are commercially produced. Live attenuated bacteria that have been shown to be highly effective as vaccine candidates however, drawbacks such as safety, reactogenicity, stability, and manufacturing remain problematic (Galen and Curtiss, 2013). Whole-cell-killed bacterial vaccines are easy to commercially manufacture, but have problems with stability, long-term protection, and present biosafety risks in the case of class III pathogens. Purified surface carbohydrates have been utilized as a vaccine candidate, but typically only produce short-term protection and are not effective in children or mature individuals (Lockhart, 2003). Traditional conjugate vaccines, where bacterial surface polysaccharides are chemically conjugated to a carrier protein, have been demonstrated to be highly effective. The best example is the *Haemophilus influenzae* type b conjugate vaccine, which has nearly eliminated infections in vast parts of the world (Pollard et al., 2009). However, manufacturing these conjugate vaccines requires complex synthetic chemistry for the attachment of the glycan to protein carriers. Additionally, the polysaccharides are either obtained from the target pathogen, which constitutes a major health hazard, or by laborious, chemical synthesis. Often, bacterial polysaccharides are too complex to be synthesized efficiently, making this process economically prohibitive. Finally, chemical attachment of the sugar to the carrier protein can result in large and heterogeneous conjugates, modifying the native structure, and thus decreasing the protective nature of the vaccine. Both live attenuated and killed bacterial vaccines have been tested against *B. pseudomallei*, but provide little to no protection against disease and mortality in murine virulence models (Peacock et al., 2012). Additionally, since *B. pseudomallei* requires class III biosafety facilities, manufacturing glycoconjugates containing glycans from its native host is challenging and possibly hazardous. Recently, it has been demonstrated a protein chemically conjugated with the *B. pseudomallei* OPS and CPS was able to increase survival against *B. pseudomallei* infection (Scott et al., 2014). Additionally, protection has been demonstrated using *B. thailandensis* E555 as a live vaccine due to homology of CPS structures (Scott et al., 2013).

A novel method of synthesizing conjugate vaccines is through the exploitation of the protein glycosylation machineries of bacteria (Iwashkiw et al., 2012; Cuccui et al., 2013; Wetter et al., 2013). The cornerstone of bacterial glycosylation is the oligosaccharyltransferase (OTase) enzymes, which covalently attach glycan structures to either asparagine (*N*-linked) or serine/threonine (*O*-linked) residues (Nothaft and Szymanski, 2010). OTases have high substrate promiscuity, and thus can transfer a wide range of glycan structures to acceptor proteins, in a process called OTase-dependent glycosylation. The best characterized *N-*glycosylation system in bacteria is from *Campylobacter jejuni* (Nothaft et al., 2010). Briefly, a unique initiating glycosyltransferase attaches a nucleotide-activated monosaccharide-1P to the lipid carrier undecaprenyl phosphate (Und-P) in the cytoplasmic face of the inner membrane. Subsequently, a series of other glycosyltransferases attach additional monosaccharides to first residue, and when completed, the lipid-linked oligosaccharide (LLO) is translocated to the periplasmic face of the inner membrane by a flippase. Finally, PglB (*N*-OTase) covalently attaches the glycan to asparagine residues with the sequon D/E-X-N-Y-S/T (X,Y ≠P; Kowarik et al., 2006). Earlier studies demonstrated PglB has relaxed glycan specificity, allowing the transfer of a vast array of glycans, including O antigens, to acceptor proteins (Feldman et al., 2005). It was later shown that *O*-OTases share this feature (Faridmoayer et al., 2008). Thus, bacterial glycosylation systems can be exploited to synthesize novel glycoconjugates for vaccination and diagnostic purposes as previously demonstrated (Ihssen et al., 2010; Iwashkiw et al., 2012; Cuccui et al., 2013; Wetter et al., 2013; Wacker et al., 2014). Glycoconjugates produced by this method are significantly less expensive, less challenging to produce, and produce less hazardous waste than conventional chemical methods.

In this work, we demonstrate that the biosynthesis of the *B. pseudomallei* OPS II can be reconstituted in *Escherichia coli* (*E. coli*). Successful generation of the conjugate required the expression of the corresponding *B. pseudomallei* OPS II genes in an *E. coli* strain lacking both the *waaL* ligase and *wecA* initiating glycosyltransferase (SDB1). This glycoconjugate, when injected into mice, was able to develop a directed IgG immune response toward *B. pseudomallei*, and provide partial protection against infection in a murine model of melioidosis.

## MATERIALS AND METHODS

### BACTERIAL STRAINS, PLASMIDS, AND GROWTH CONDITIONS

*Escherichia coli* strains were grown on LB broth at 37°C. Trimethoprim (100 μg/ml), spectinomycin (80 μg/ml), and ampicillin (100 μg/ml) were added to the media for plasmid selection as needed. The strains and plasmids used are listed in **Table 1**.

**Table 1:** List of strains and plasmids utilized.

Strain	Genotype or description	Reference
EPI300	F-mcrA Δ(mrr-hsdRMS-mcrBC) ϕ80dlacZΔM15 ΔlacX74 recA1 endA1 araD139 Δ(ara, leu)7697 galU galK λ-rpsL (StrR) nupG trfA dhfr	Epicentre
Top 10	F-mcrA Δ(mrr-hsdRMS-mcrBC) φ80lacZΔM15 ΔlacX74 nupG recA1 araD139 Δ(ara-leu)7697 galE15 galK16 rpsL(Str^R^) endA1 λ^-^	Invitrogen
CLM24	W3110, Δ*waaL* ligase	Feldman et al. (2005)
CLM37	W3110, ΔwecA	Linton et al. (2005)
BW25113 rfe::kan	F-Δ(araD-araB)567 ΔlacZ4787(::rrnB-3) LAM-rph-1 Δ(rhaD-rhaB)568 hsdR514	Baba et al. (2006)
SDB1	W3110, Δ*waaL* ligase, Δ*wecA* GalNAc transferase	This study
**Plasmids**
pBAD24	Cloning and expression vector, Arabinose inducible, Amp^R^	Guzman et al. (1995)
pEXT21	Cloning and expression vector, IPTG inducible, Sp^R^	Dykxhoorn et al. (1996)
pMLBAD	Cloning and expression vector, Arabinose inducible, Tp^R^	Lefebre and Valvano (2002)
pEQ3	*B. pseudomallei* type II OPS, Ap^R^	This study
pIH18	Soluble periplasmic *C. jejuni* acrA_6xHis_ cloned into pEXT21, Sp^R^	Hug et al. (2010)
pMAF10	*C. jejuni pgl*B cloned into pMLBAD, Tp^R^	Feldman et al. (2005)
pFLP2	Source of Flp recombinase, Ap^R^	Hoang et al. (1998)
pCA24N-waaL	*E. coli waaL* cloned into pCA24N from ASKA library; Cm^R^	Kitagawa et al. (2005)
pCA21	*E. coli wecA* cloned into pEXT21, Sp^R^	Alaimo et al. (2006)
pJHCV32	HindlIl cosmid clone in pVK102, 07+ Tcr	Valvano and Crosa (1989)
pCC1FOS-BPF16β_E10	LPS cluster of *B. pseudomallei* K96243, coordinates 3191324-3229257	Titball Lab (unpublished)

### WESTERN BLOTTING

Glycan expression and glycosylation were analyzed by SDS-PAGE on 10% gels. The gels were electroblotted onto a nitrocellulose membrane via semi-dry membrane transfer and analyzed with antibodies α-His (Santa Cruz Biotechnology) and α-BPs OPSII glycan kindly provided by Dr. Joanne Prior (1:1,000). Membranes were visualized using the Odyssey Infrared Imaging System (Li-Cor Biosciences, USA).

### CLONING AND EXPRESSION OF *Burkholderia pseudomallei* TYPE II OPS

To obtain the plasmid expressing the type II O-antigen polysaccharide of *Burkholderia pseudomallei* under an arabinose promoter we used the pCC1FOS-BPF16β_E10 vector kindly provided by Professor R. Titball (University of Exeter), which contains the LPS cluster of *B. pseudomallei* K96243, coordinates 3191324–3229257. The pCC1FOS-BPF16β_E10 vector was digested with *Nhe*I, *Kpn*I, and *Pci*I to get an 8673 bp fragment, corresponding to genes between *rmlD* and *wbiC*, and with *Sna*BI and *Kpn*I to get a 9367 bp fragment that includes the genes between *wbiC* and *wbiI*. These two fragments containing the 15 genes required for *B. pseudomallei* type II OPS expression were inserted into pBAD24 digested with *Nhe*I and *Sma*I. Arabinose-dependent expression of the type II O-antigen was confirmed by Western blot.

### CONSTRUCTION OF SDB1 *waaL* AND *wecA* DEFICENT STRAIN

Construction of SDB1 strain was done using the P1 transduction protocol adapted from Thomason et al. (2007). The P1 bacteriophage was first grown on the strain (BW25113 *rfe::kan*.) from the Keio collection library (Baba et al., 2006). This strain has a kanamycin-resistant cassette on the *wecA* gene as a donor. The resulting phage lysate was used to infect the recipient strain CLM24 (Δ*waaL*). Recombinant strains were confirmed by PCR using the oligonucleotides rfe for comp (5′-GCAATGACCAAGACCAATGACG-3′) and rfe rev comp (5′-GCTGCTGCGAGTAATATCCC-3′). The kanamycin cassette was removed using the FLP recombinase expressed from pFLP2.

### PRODUCTION AND PURIFICATION OF GLYCOSYLATED AcrA

SDB1 strain transformed with *C. jejuni* PglB (pMAF10), AcrA (pIH18), and BPs type II O-antigen (pEQ3) was grown overnight at 37°C. Culture was reinnoculated 1/33 into fresh LB media using a culture/flask ratio 1:10. After 2 h at 37°C with shaking at 200 rpm, the cultures were induced with 0.1 mM isopropyl 1-thio-β-D-galactopyranoside (IPTG; Sigma) and 0.2% (w/v) L-(+)-arabinose (MP Biomedicals). To increase the glycosylation yield in SDB1, we also added MnCl_2_ (4 mM). Five hours after induction at 37°C, arabinose was added again to ensure PglB expression. Cells were harvested by centrifugation after an overnight induction period and the periplasmic extract containing the glycoproteins was extracted using a lysozyme treatment as described previously (Iwashkiw et al., 2012). For purification, the periplasmic fraction was equilibrated with 1/9 vol 10× loading buffer (0.1 M imidazole, 3 M NaCl, 0.2 M Tris-HCl, pH 8.0) and subjected to a Ni^2+^affinity chromatography as described (Iwashkiw et al., 2012). Purified protein was quantified by Bradford assay (BioRad).

### SUGAR QUANTIFICATION OF GLYCOPROTEINS

The protocol was adapted from Dubois et al. (1956). In a small glass tube was mixed 10 μl of sample, 90 μl of ddH_2_O, and 100 μl of freshly made 5% phenol in ddH_2_O. Then 1 ml of concentrated H_2_SO_4_ was briskly added into the mixture and immediately vortex for several seconds. An orange color with intensity proportional to concentration began to develop and reached a maximum about 2 h at 30°C. The samples were read against glucose standards at OD_500._

### VACCINATION

BALB/c mice (*n* = 5 per group, 6-week-old female) were immunized with three doses of purified recombinant bioconjugate vaccine, carrier protein, or gamma-irradiated killed whole cells (3 × 10^4^ *Burkholderia pseudomallei* K96234), via the intraperitoneal route (i.p.) over 6 weeks. The doses were administered with Imject Alum Adjuvant (Thermo Scientific), not used with whole killed cells or when noted. Sera samples were collected for antibody analysis, prior to immunization 2 weeks after vaccination and boost. The antibody titre of total IgG was analyzed by ELISA. Briefly, wells of microtiter plates were coated (18 h, 4°C) with gamma-irradiated whole *Burkholderia pseudomallei* cells at a 1/100 dilution in 100 μl of coating buffer (0.05 M Na_2_CO_3_, 0.05 M NaHCO_3_, pH 9.6) and were then blocked with 2% (w/v) BSA in PBS for 2 h at 37°C. Sera samples at a 1/200 dilution in 100 μl of antibody dilution buffer [2% (w/v) BSA, 0.1% (v/v) Tween 20] were incubated for 1 h at 37°C. HRP-conjugated goat anti-mouse IgG at a 1/8000 in antibody dilution buffer was added for 1 h at 37°C and then the reaction was visualized by the addition of 100 μl chromogenic substrate (ultra-TMB) for 5 min. The reaction was stopped with 100 μl H_3_PO_4_ and absorbance at 405 nm was measured using ELISA plate reader. Plates were washed five times with washing buffer [0.1% (v/v) Tween 20] after each step.

### INTRANASAL CHALLENGE MODEL

The murine melioidosis infection model used was carried out under ABSL-3 containment practices. Briefly, female BALB/c mice were challenged via the i.n. route (50 μl) with approximately 2 × 10^3^ CFU (approximately 10–12 LD_50_) of *B. pseudomallei* K96243. Mice were weighed prior to inoculation and monitored for 21 days post-infection. Mice were anesthetized, held vertically, and 50 ml of the inoculum was released into the nares for inhalation. Following challenge, the inoculum was back titrated on agar plates to confirm delivered dose. Using this model, control mice died or were euthanized according to predetermined humane end points 3–6 days post-challenge.

### STATISTICS

Survival curves were generated by use of Kaplan–Meier estimators. The survival distributions of each treatment group vs. control protein carrier group were compared by unpaired *T* test or Mann–Whitney test using GraphPad Prism version 6.0.

### ETHICS STATEMENT

This study was carried out in accordance with the Canadian animal care guidelines. The protocols were approved by the Animal Care and Use Committees of Defence Research and Development Canada. Mice were anesthetized by intraperitoneal injection of a sodium pentobarbital solution.

## RESULTS

### CLONING AND EXPRESSION OF THE *B. pseudomallei* k96243 O ANTIGEN POLYSACCHARIDE II (OPS II) LOCUS IN *E. Coli*

**FIGURE 1:**
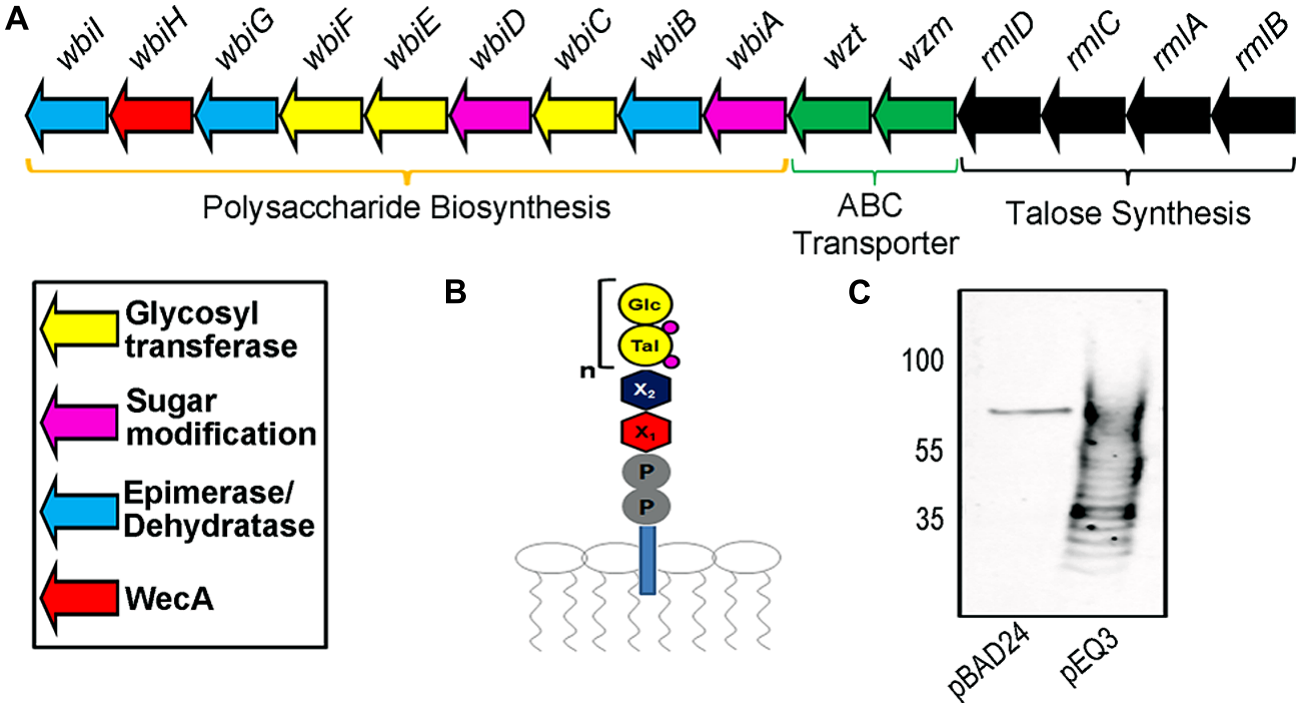
**Description of *Burkholderia pseudomallei* O-polysaccharide II (OPSII) cluster. (A)** Representation of the required 15 genes for the synthesis of OPS II and predicted functions. **(B)** Expected glycan intermediate structure attached to Undecaprenyl pyrophosphate. **(C)** Western blot of 10 μl of whole-cell extracts utilizing an anti-Glycan antibody reveals expression of OPS II (subcloned in pEQ3) in *E. coli* produces a polymer in whole-cell extracts.

Previous work identified a region consisting of 21 potential open reading frames, and further investigation identified a cluster of 15 genes required for the biosynthesis of *B. pseudomallei* K96243 OPS II (**Figure 1A**; DeShazer et al., 1998). A previous study demonstrated by NMR analysis that the structure of OPS II is a polymer of a disaccharide repeating structure composed of -3-)-β-D-glucopyranose-(1–3)-α-L-6-deoxy-talopyranose-(1-, with variable *O-*methyl and *O*-acetyl modifications (**Figure 1B**; Perry et al., 1995). In order to recombinantly express the *B. pseudomallei* OPS II in *E. coli*, the 15 essential genes were subcloned (genes *rmlB* to *wbiI*) from the plasmid pCC1FOS-BPF16β_E10 by restriction digest into the arabinose inducible expression vector pBAD24, generating pEQ3 (**Figure 1A**). Expression of the *B. pseudomallei* OPS II in *E. coli* CLM37 was visualized by Western blot as a typical ladder of immunoreactive bands, confirming the production of the carbohydrate structure (**Figure 1C**).

### GENERATION OF AN *E. Coli* STRAIN OPTIMIZED FOR OPS II PRODUCTION AND PROTEIN GLYCOSYLATION

We attempted to generate a *N*-linked glycoprotein with the OPS II by exploiting the *C. jejuni N-*glycosylation system as previously described (Ihssen et al., 2010; Iwashkiw et al., 2012; Cuccui et al., 2013; Wetter et al., 2013). In earlier work, *N*-glycosylated AcrA was synthesized in *E. coli* by co-expression of *C. jejuni* PglB and AcrA with an appropriate carbohydrate structure. We therefore expressed PglB (pMAF10), AcrA (pIH18), and the *B. pseudomallei* OPS II antigen (pEQ3) in both a traditional expression (EPI300) and *wecA-* (CLM37) strains and tested for glycosylation by Western blot. We were unable to detect any evidence of glycosylation of purified AcrA (data not shown).

**FIGURE 2:**
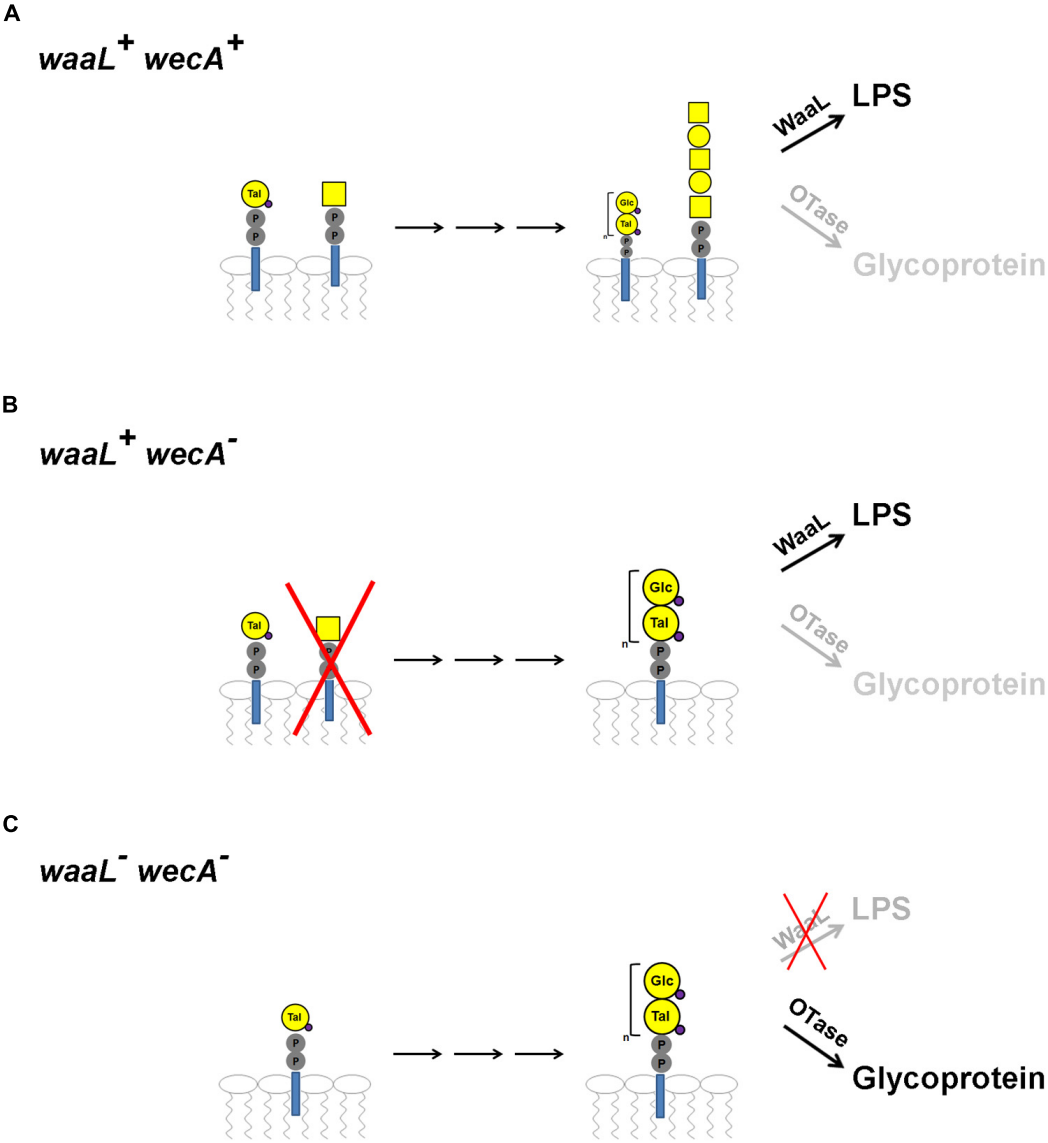
**Hypothesis for the lack of glycosylation due to glycan competition and utilization, resulting in the development of the *E. coli* strain SDB1. (A)** When expressing pEQ3 in a WT *E. coli* background, OPS II is produced at a lower quantity than the native O-antigen, and both structures are preferentially transferred by WaaL to Lipid A, instead of to the acceptor protein by PglB (OTase). **(B)** If *wecA* is deleted, the native O-antigen cannot be produced, allowing for a greater production of OPS II. However, OPS II would be still selectively transferred by WaaL over PglB. **(C)** If both *wecA* and *waaL* are deleted, the OPS II structure would be highly produced, and only available to PglB for synthesis of glycoproteins.

One issue with exploiting O antigens using protein glycosylation may be the precursor can also be used by the WaaL ligase in *E. coli*, thus siphoning off the substrate, and decreasing the glycosylation efficiency (**Figure 2**). Additionally, *E. coli* strains express the initiating glycosyl transferase (*wecA*), which transfers a GlcNAc onto the undecaprenyl-diphosphate (Und-PP) carrier. This would interfere with the synthesis of the glycan of interest onto the same lipid if the first sugar in the structure is not a GlcNAc, as in the case of the OPS II. We hypothesized that deletion of WaaL and WecA would result in an increased efficiency of protein glycosylation (**Figure 2C**).

**FIGURE 3:**
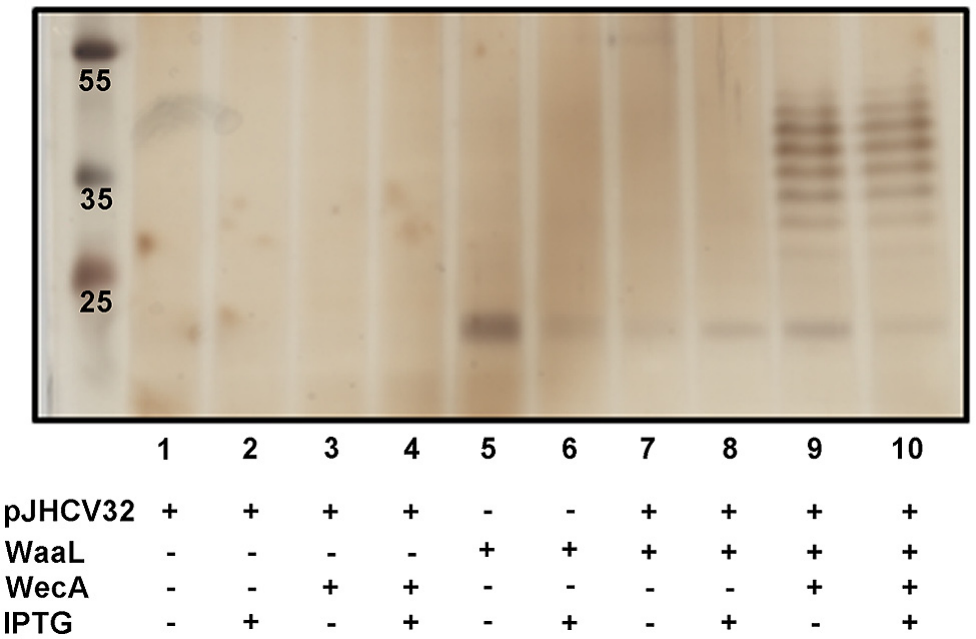
**Confirmation of a *wecA- waaL-* double mutant (SDB1) in *E. coli* W3110.** To verify the double mutant, LPS extractions (20 μl per sample) were analyzed by silverstain for the ability to produce exogenously expressed *E. coli* O7 LPS (pJHCV32). No LPS was observed when either WecA or WaaL was expressed *in trans* (lanes 1–8). However, when both were coexpressed, a laddering pattern was observed, confirming complementation of the double mutant (lanes 9 and 10).

We therefore constructed the *E. coli* *wecA^-^ waaL^-^* mutant strain SDB1. Using the KEIO strain collection (Baba et al., 2006), the *wecA* mutation was transduced into CLM24, an *E. coli waaL* mutant (Feldman et al., 2005), creating SDB1. To functionally confirm the double mutation, we analyzed the LPS produced by SDB1 transformed with plasmid pJHCV32 (**Figure 3**). The plasmid pJHCV32 drives the constitutive expression of the *E. coli* O7 antigen, but relies on the chromosomal copy of the glycosyltransferase WecA (Valvano and Crosa, 1989). Expression *in trans* of either WecA or WaaL individually in this background did not result in the production of a smooth LPS containing the O7 antigen (lanes 1–8). However, when both genes were co-expressed in SDB1, we observed the characteristic polymerization of O antigen previously observed for the *E. coli* O7 LPS (lanes 9 and 10), confirming the creation of the double mutation.

### *IN VIVO* SYNTHESIS AND PURIFICATION OF AN *N-*LINKED GLYCOCONJUGATE WITH THE *Burkholderia* OPSII GLYCAN

**FIGURE 4:**
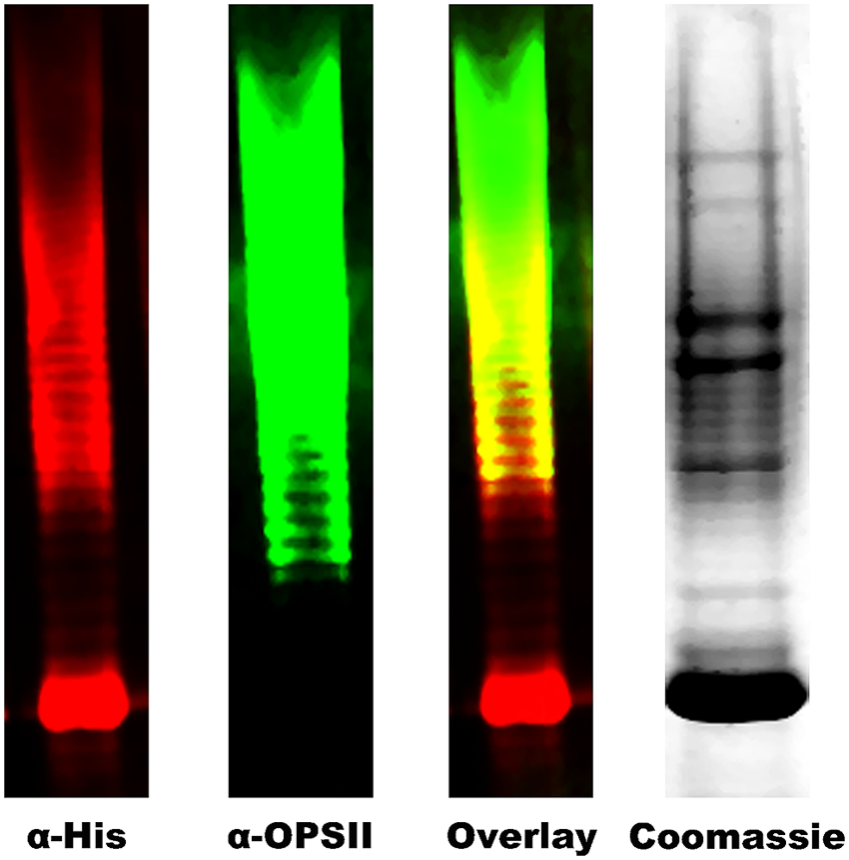
**Analysis of purified AcrA by Western Blot.** SDB1 transformed with pMAF10 (*pglB*), pIH18 (*acrA*), and pEQ3 (OPS II) were grown (+/-) induction of OPS II, harvested, and AcrA was purified by Ni^2+^-NTA affinity chromatography. Purified glycoconjugate was separated on 10% SDS-PAGE and analyzed by western blot (5 μl) or Coomassie stain (15 μl). A high-molecular-weight ladder is co-detected by both α-His and α-Glycan antibodies, and overlap of the two signals strongly suggests glycosylation of AcrA. Staining of the purified protein is observed by Coomassie stain.

To create an *N-*linked glycoconjugate, we transformed the *E. coli* strain SDB1 with the pEQ3 (OPS II), pMAF10 (PglB), and pIH18 (AcrA). Cultures of the transformed strain were grown and induced as required, and AcrA was purified from periplasmic extracts by Ni^2+^ affinity chromatography. To determine if AcrA was glycosylated, we analyzed the purified protein by either Western blot with antibodies specific to either AcrA or OPS II, or Coomassie stain, and when visualized together, we observed an overlap of the signal, suggesting glycosylation of AcrA with the OPSII glycan (**Figure 4**).

**FIGURE 5:**
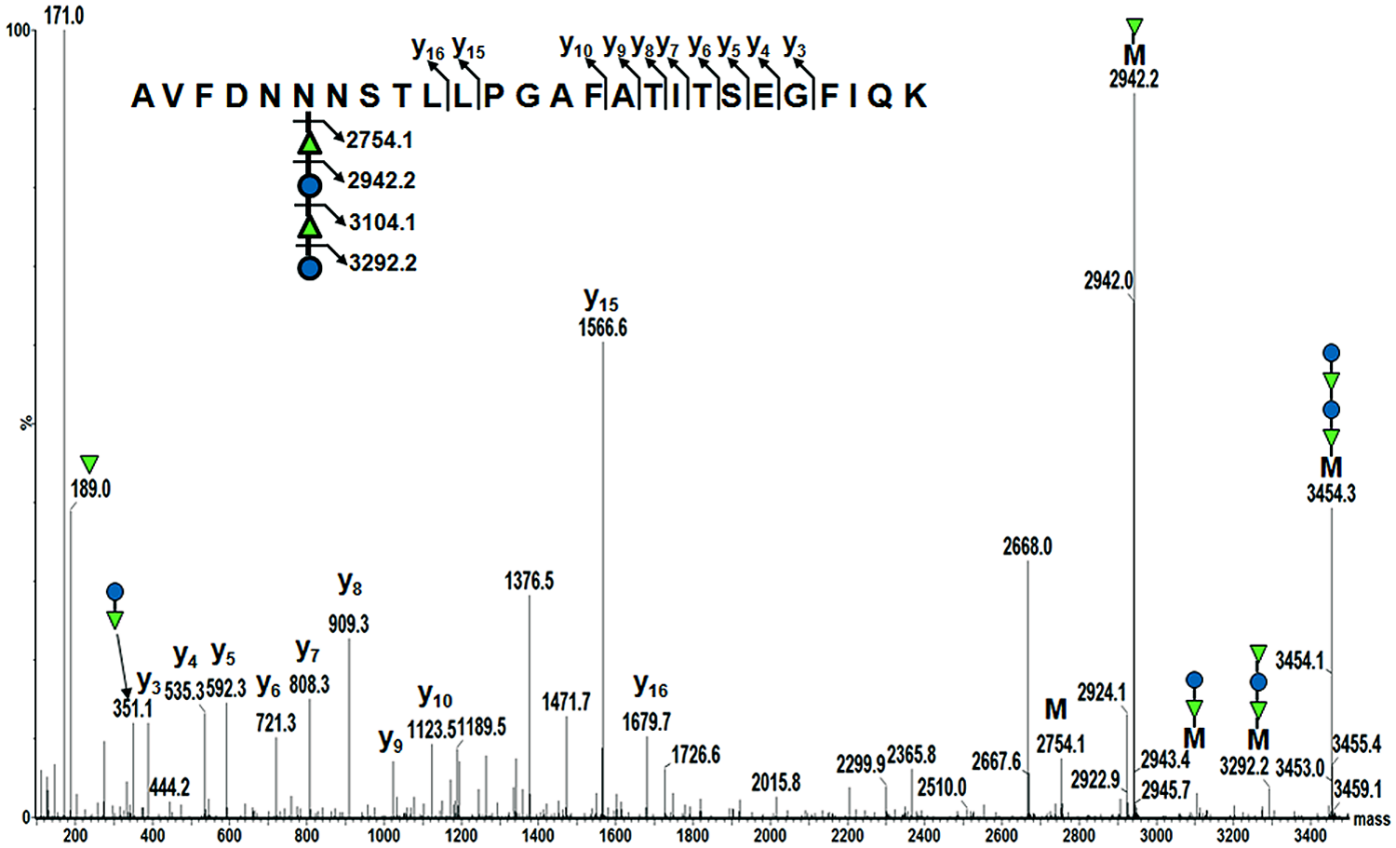
**Mass spectrometry identification of the *B. pseudomallei* OPS II glycan attached to AcrA.** MS analysis of tryptically digested glycosylated AcrA revealed a peak of *m/z* 1152.06^3+^. MS/MS of this peak showed a previously characterized glycosylation site of AcrA (AVFDNNNSTLLPGAFATITSEGFIQK; 2754.1 Da) with a modification of 700.2 Da. Analysis of the modification revealed a tetrameric glycan, with a structure of 188–162–188–162. This structure is consistent with the previously determined structure of *B. pseudomallei* OPS II being a polymer of dimers of *O*-acetylated deoxytalose (188 Da) and glucose (162 Da).

To confirm AcrA was glycosylated with the *B. pseudomallei* OPS II carbohydrate, we employed mass spectrometry (MS) techniques. The purified glycoprotein was tryptically digested in-solution, and the resulting peptides were examined by LC-ESI-Q-TOF MS and MS/MS. Manual analysis of the MS data (MassLynx; Waters Corporation) revealed a peak with an *m/z* 1152.06^3+^, and further inspection of this peak by MS/MS revealed a glycopeptide that corresponded to the previously identified second glycosylation site of AcrA (AVFDNNNSTLLPGAFATITSEGFIQK; *m/z* 2754.1) with the addition of an *m/z* 700.2 modification (**Figure 5**). *De novo* peak annotation identified the modification to be a tetramer of 188–162–188–162. The mass of 188 Da is consistent with an *O*-acetyl deoxyhexose residue, and the 162 Da is consistent with a hexose residue. We were also able to identify in the low-molecular region both an individual *O*-acetyl deoxyhexose (189.0 Da), and a subunit of the dimer with a mass of 351.1 Da. Our MS characterization of the *B. pseudomallei* OPS II glycan is consistent with the previously published data identifying it to be a polymer of dimers of *O*-acetylated deoxytalose and glucose (Perry et al., 1995). These data combined with the immunoreactivity of our glycoconjugate to the *B. pseudomallei* OPS II antibody confirm that we were able to synthesize and glycosylate AcrA with the correct glycan structure in *E. coli* SDB1.

### MICE INJECTED WITH PURIFIED *N-*LINKED BIOGLYCOCONJUGATE DEVELOPED A PARTIALLY PROTECTIVE IgG IMMUNE RESPONSE TOWARD *B. Pseudomallei* WHOLE CELLS

**FIGURE 6:**
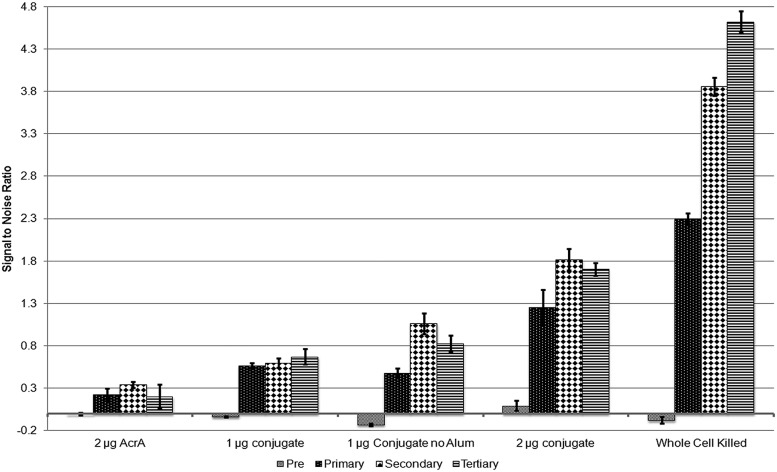
**ELISA of the IgG immune response of immunized mice toward whole-cell extracts of *B. pseudomallei.*** All data were normalized to the PBS-injected control mice, and no response was observed in all groups prior to immunization. A low level of response was observed at a consistent level for the unglycosylated AcrA control, whereas all test groups injected with glycosylated AcrA had a significant IgG response. The results are the median of five mice in each test group.

To evaluate the potential use of the glycoprotein as conjugate vaccine, the purified AcrA containing OPS II was injected intraperitoneally into mice to measure the immune response compared to whole-cell-killed *B. pseudomallei*. Groups of five mice were injected with PBS, unglycosylated AcrA as control, glycoconjugate in different quantities, or whole-cell-killed cells. The IgG immune response was tested by ELISA against whole-cell extracts of *B. pseudomallei* (**Figure 6**). All test groups were compared to the PBS control, and showed no initial immune response toward *B. pseudomallei* in the pre-injection sera. The AcrA-injected group had a slight increase in IgG response, but did not increase after additional boosts. A significant increase in IgG response was observed in each of the glycosylated test groups after the primary vaccination, with varying degrees of improvement in immune response after a second and third boost. The best immune response was observed in the mice injected with whole-cell lysates of *B. pseudomallei* had a significantly stronger immune response as compared to the glycoconjugate sera, but this was expected as whole cells were use as antigen for the ELISA.

**FIGURE 7:**
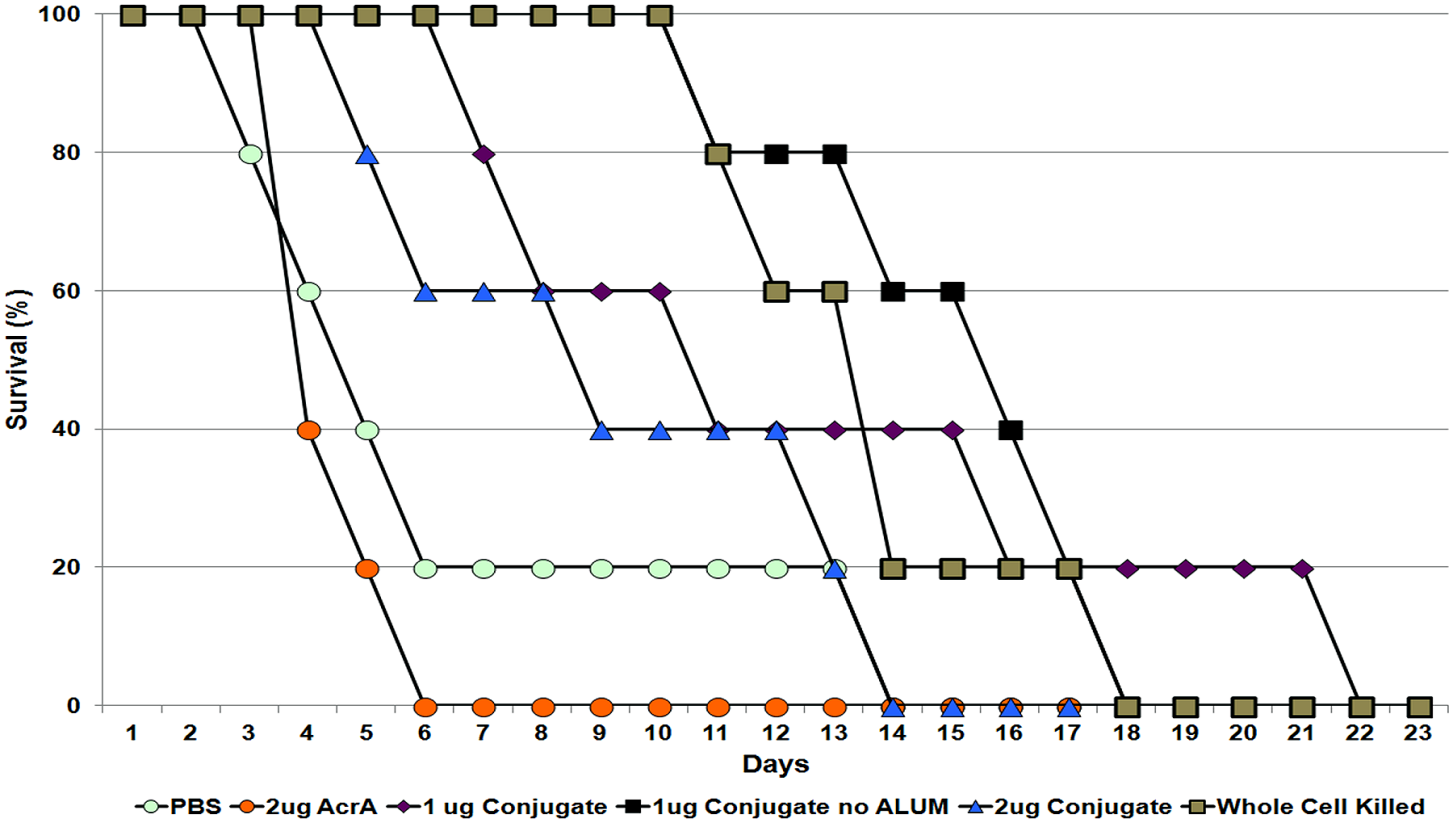
**Murine survival of different test groups to a 12× LD_50_ i.n. challenge of *B. pseudomallei*.** Mice vaccinated with either PBS or unglycosylated AcrA controls had a significantly lower survival period as compared to the mice injected with OPS II glycosylated AcrA. Interestingly, mice vaccinated with 1 μg glycoconjugate (+/- adjuvant) had a higher survivorship percentage as compared to the 2 μg vaccinated group.

We next tested the immunized mice for a preliminary evaluation of the efficacy of the glycoconjugate. We employed an intranasal murine melioidosis model against *B. pseudomallei* infection with a dose of 12× LD_50_ (**Figure 7**). Mice vaccinated with only protein carrier died or were euthanized according to predetermined humane end points after 6 days. For the PBS-injected control group, 80% of the mice died or were euthanized after 6 days, and one mouse survived until day 13 of the challenge. All of the mice vaccinated with the glycoconjugate showed a significant increase in survival time as compared to the control protein carrier group. However, contrary to the ELISA results that showed the best IgG immune response in mice vaccinated with 2 μg of glycosylated AcrA, 40% of the mice survived until day 12, with the remaining being sacrificed on day 14. Mice injected with 1 μg of glycoprotein without any adjuvant saw survival until day 18, while one mouse vaccinated with 1 μg survived until day 22. The difference in mean time to death was not statistically significant between the groups receiving the various glycoprotein preparations. In comparing our glycoconjugate to whole-cell-killed bacteria as vaccine candidates, we observed highly similar survival of the mice, with all mice succumbing to infection by day 18 of the challenge. Overall, these results demonstrate that the *N-*glycoconjugate containing the *B. pseudomallei* OPS II is capable of providing partial immune protection against a 12 × LD_50_ dose, and it is comparable to whole-cell-killed bacteria in protection against infection.

## DISCUSSION

Due to a combination of factors including the increasing number of reported cases of *B. pseudomallei* infections, the risk posed by the bacterium as a potential biological warfare agent, and an absence of an effective vaccine, we explored the possibility that by exploiting the *N*-linked glycosylation system of *C. jejuni*, we could produce a glycoconjugate vaccine containing the OPS II of *B. pseudomallei.* This strategy was only demonstrated in a few cases (Iwashkiw et al., 2012; Cuccui et al., 2013; Wetter et al., 2013). Since *B. pseudomallei* is a biosafety class III agent we were unable to directly exploit the native OPS II by expressing the *N*-glycosylation system in the host as previously shown (Iwashkiw et al., 2012). Instead, we utilized previous knowledge of the genetic loci responsible for the biosynthesis of OPS II (DeShazer et al., 1998), and subcloned the key 15 genes into an *E. coli* expression vector. We observed high levels of expression of the OPS II in *E. coli* strains by Western blot, and attempted to create the glycoconjugate in a basic expression and *wecA- E. coli* strains as previously described (Ihssen et al., 2010; Cuccui et al., 2013; Wetter et al., 2013). We did not observe any evidence of glycosylation, and hypothesized that the OPS II was being utilized exclusively to modify the LPS. Therefore, we engineered an *E. coli* strain (SDB1) lacking both the *waaL* ligase, and *wecA*. Construction of the SDB1 strain was confirmed by expressing either or both WecA and WaaL with a plasmid encoding the *E. coli* O7 LPS cluster that lacks WecA homolog. Analysis of LPS extractions by silverstain showed that the O7 antigen structure was only transferred to lipid A in the presence of both enzymes. Our engineered strain in theory should be able to produce the OPS II-AcrA glycoconjugate with high efficiency due to no other competition for either the undecaprenyl-phosphate lipid carrier by WecA or the OPS II-Und-PP substrate from WaaL.

We showed that SDB1 produced the desired glycoconjugate when OPS II (pEQ3), PglB (pMAF10), and AcrA (pIH18) were co-expressed. The generation of the conjugate was shown via Western blot and MS. Interestingly, glycosylation of AcrA required the addition of MnCl_2_ to the media. Although Mn^2+^ is required for PglB activity and was observed in the active site of the crystal structure (Lizak et al., 2011), we did not have to add Mn^2+^ to obtain glycosylated proteins in previous experiments. The direct relationship between its addition and glycosylation efficiency is unclear. The MS examination of the purified glycoconjugate revealed the presence of a tetrameric glycan moiety corresponding to two repeats of *O*-acetylated deoxyhexose and hexose, in agreement with previously published characterization of the structure (Perry et al., 1995). Interestingly, previous work has shown that the *C. jejuni* OTase PglB can only transfer glycans with a reducing monomer with an *N*-acetyl group, whereas the *B. pseudomallei* OPS II structure has been shown to possess an *O*-acetyl modification. This finding expands our knowledge on the substrate specificity of PglB. We did not observe any larger glycan structures that were detected by Western blot, but this could be due to limitations of our MS instruments. Most of the Wzy-independent glycans require an adapter composed of two monosaccharides linking the lipid carrier to the polymeric structure (Whitfield, 2006; Greenfield and Whitfield, 2012). The finding that the *O*-acetylated deoxytalose appears to be directly linked to the protein indicates that a linker glycan (proposed in **Figure 1A** as X1 and X2) is not present in the OPS II structure. Our experiments demonstrate that in the genetically engineered strain SDB1, we were able to produce a glycoconjugate with the *B. pseudomallei* OPS II carbohydrate.

We carried out preliminary experiments to determine if our glycoconjugate could be utilized as a vaccine against *B. pseudomallei.* We injected different quantities of the glycoconjugate into mice. We observed virtually no response toward *B. pseudomallei* for the mice injected with the protein carrier alone, whereas a significantly stronger response from the mice injected with all groups of the glycoconjugate. Interestingly, an intermediate response was observed in the 1 μg glycoconjugate groups, regardless of the addition of an adjuvant. We then challenged the vaccinated mice with *B. pseudomallei* K96243, and after 6 days post challenge, none of the carrier control injected mice survived, whereas survival was observed each of the glycoconjugate groups, with the longest survival in the 1 μg group, irrespective of the addition of an adjuvant. Why lower levels of antibody production resulted in better protection remains unknown, although these differences in protection were not statistically significant between groups receiving the glycoprotein. It is possible that the carrier protein, AcrA, due to its high immunogenic nature, acts as an adjuvant itself. This could also possibly explain why both of the 1 μg groups while having a lower detected IgG immune response compared to the 2 μg group have a longer survival period. Our results are consistent with these previous studies with the non-protected mice succumbing to infection after ∼1 week (Nelson et al., 2004; Su et al., 2010; Nieves et al., 2011). Interestingly, our initial studies with the glycoconjugate gave similar protection levels to whole-cell-killed *B. pseudomallei*. Further optimization of our vaccine candidate is currently undergoing. This includes testing different amounts of conjugate, and replacing the acceptor protein from AcrA of *C. jejuni* to a *B. pseudomallei* protein, which may enhance the immunogenicity of the conjugate.

Several other studies have been published attempting to develop a vaccine against *B. pseudomallei* (reviewed in Silva and Dow, 2013). Many of these vaccine candidates have dealt with either attenuated strains, whole-cell-killed bacteria, or purified proteins directly from *B. pseudomallei*, which requires class III biosafety facilities, and would lead to higher significant problems for commercialization of the product. Other groups attempted to recombinantly express and purify *B. pseudomallei* proteins in *E. coli*, with limited success. However, our work has demonstrated that the OPS II carbohydrate of *B pseudomallei* can be functionally expressed in *E. coli*, and be utilized by PglB to create a glycoconjugate that is partially protective against infection. Glycoengineered therapeutics are simple to produce, cost effective, and have been demonstrated to provide long-term protection against several pathogens. Additionally, previous work has used a similar glycoconjugate with the *Y. enterocolitica* O9 O-antigen as a diagnostic tool (Iwashkiw et al., 2012). Conjugation of the glycoconjugate to magnetic nanobeads has shown significant promise in detection of disease in cattle and human sera samples (Ciocchini et al., 2013, 2014). A similar system may have future potential for diagnosing individuals infected with *B. pseudomallei.*

In summary, we have demonstrated that the OPS II glycan of *B. pseudomallei* can be functionally expressed in *E. coli*. Additionally, this glycan was transferred to the carrier protein AcrA by the OTase PglB, both from *C. jejuni*, to generate a glycoconjugate. We also described a novel *E. coli* strain SDB1 which lacks *wecA* and *waaL,* resulting in a higher efficiency of glycosylation as compared to previously used strains. Mice injected with this glycoprotein were able to develop a long-term IgG immune response, and showed significantly longer survival when challenged with *B. pseudomallei* as compared to the naive controls. This new biologically engineered strain may be used for the future creation of commercial bioglycoconjugate therapeutics, and glycoconjugate may have future potential for diagnostic applications or vaccination against *B. pseudomallei* infections.

## Conflict of Interest Statement

The authors declare that the research was conducted in the absence of any commercial or financial relationships that could be construed as a potential conflict of interest.
